# Management of Bullous Pemphigoid and Norwegian Scabies with Dupilumab Plus Ivermectin

**DOI:** 10.4269/ajtmh.26-0186

**Published:** 2026-09-22

**Authors:** Yanli Zhang, Yuting Liu, Hengan Yi

**Affiliations:** ^1^ Department of Dermatology, The Third People’s Hospital of Longgang, Clinical Institute of Shantou University Medical College (The Third People’s Hospital of Longgang District Shenzhen), Shenzhen, China;; ^2^ Department of Dermatology, Shenzhen People’s Hospital (The First Affiliated Hospital, Southern University of Science and Technology, The Second Clinical Medical College, Jinan University), Shenzhen, China;; ^3^ Department of Dermatology, Shenzhen Maternity and Child Healthcare Hospital, Southern Medical University, Shenzhen, China

## Abstract

Bullous pemphigoid is a severe autoimmune blistering disease, and Norwegian scabies is a rare comorbidity. However, conventional treatment methods, such as systemic corticosteroids, carry a high risk in the treatment of elderly patients. Herein, this rare comorbidity is described in an elderly patient. After an initial induction treatment with a low-to-moderate dose of corticosteroid, this patient achieved complete remission with a combination therapy of dupilumab (a monoclonal antibody targeting the interleukin-4/13 signaling pathway) and ivermectin. Therefore, the successful treatment of this rare case provides a promising strategy for reducing reliance on corticosteroids in high-risk populations.

## INTRODUCTION

Bullous pemphigoid (BP) is an autoimmune subepidermal blistering disorder that predominantly affects the elderly.^1^^–^^4^ Norwegian scabies (NS) is a severe cutaneous infestation caused by *Sarcoptes scabiei* (var. *hominis*), marked by hyperkeratotic crusting plaques.^5^^–^^7^ The concurrence of NS and BP is relatively rare and may be attributed to immunosuppressant use and advanced age.^8^^–^^10^ Although systemic corticosteroids are the first-line treatment of BP, they carry a high risk of severe adverse effects in elderly patients^1^^,^^11^ and are strongly associated with high mortality.^12^^,^^13^ Notably, clinical evidence confirmed that the interleukin (IL)-4/IL-13 inhibitor dupilumab enables early discontinuation of glucocorticoids, thereby reducing mortality in BP patients.^12^^–^^16^ Despite previous cases involving the coexistence of BP and NS,^8^^–^^10^ effective management strategies for geriatric patients remain suboptimal.^1^^,^^11^ Herein, a successfully managed case of NS with dupilumab and ivermectin is described, providing a viable alternative to conventional high-risk regimens.

## CASE REPORT

In September 2025, a 76-year-old man presented to the study department with a 5-month history of generalized erythema, tense bullae, and severe pruritus. His medical history included hypertension, post-cerebral infarction with bedridden status, aphasia, and malnutrition. Five months before presentation, he initially experienced symptoms of localized erythema and blisters. His family mistook the lesions for decubitus ulcers and tried to treat them with herbal preparations, which were ineffective. Additionally, the hyperkeratotic crusting on his hands worsened. Two months before presentation, the patient was suspected of pemphigoid with eczematous changes by an external hospital and received systemic corticosteroids and immunoglobulin. Although this regimen partially controlled his truncal lesions, his upper limb involvement continued to progress. Subsequently, the patient was suspected of having scabies and treated with sulfur ointment, which was ineffective. Additionally, he developed a pulmonary infection during his hospitalization. Three days post-discharge, he experienced a severe relapse involving ∼50% of his body surface area, prompting his visit to the study department.

On admission, the patient was hemodynamically stable. Cutaneous examination revealed widespread erythema and tense bullae on his head, face, torso, and lower limbs (Figure 1A), along with dry, bark-like hyperkeratotic crusts on his hands (Figure 1C). Hematoxylin and eosin staining revealed a subepidermal blister cavity filled with neutrophils, and the superficial dermis exhibited edema, mucoid degeneration, and infiltration by lymphocytes and neutrophils (Figure 2A). Periodic acid-Schiff staining revealed fragmentation of the basement membrane zone (BMZ) and a faintly positive line at the base of the blister (Figure 2B). Immunohistochemical analysis revealed positive expression of type IV collagen (Figure 2C). These findings confirm a subepidermal blister consistent with BP.^13^^,^^17^^,^^18^ Gram staining revealed no evidence of bacteria (Figure 2D). Direct immunofluorescence revealed linear deposition of C3 and IgG at the BMZ, whereas IgM, IgA, and fibrinogen results were negative (Figure 2E and F). These findings are consistent with BP.^17^ Microscopic findings revealed a scabies mite (Figure 3). Serology via ELISA confirmed elevated anti-BP230 (77.93 U/mL; ref. <20) and anti-BP180 (>400 U/mL; ref. <20) antibody levels, consistent with BP.^13^^,^^17^^,^^18^ Meanwhile, anti-desmoglein 1/3 antibody results were negative, which ruled out pemphigus vulgaris.^18^ Collectively, a final diagnosis of BP with NS was established.

**Figure 1.**
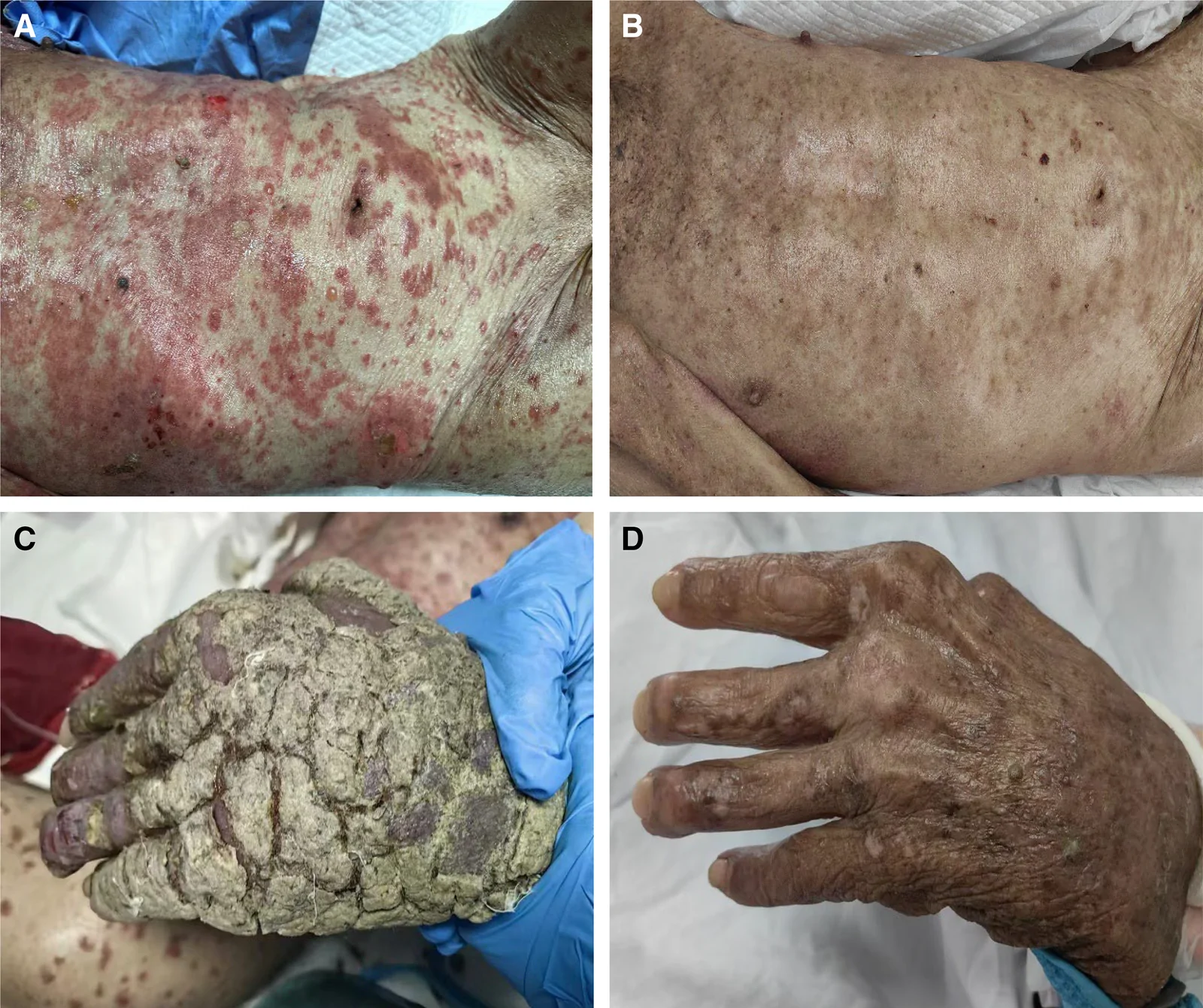
Patient outcomes before and after the intervention. Clinical presentation of (**A**) truncal and (**C**) hand lesions before intervention, along with resolution of skin lesions on the (**B**) trunk and (**D**) hands after 30 days of treatment with dupilumab and ivermectin.

**Figure 2.**
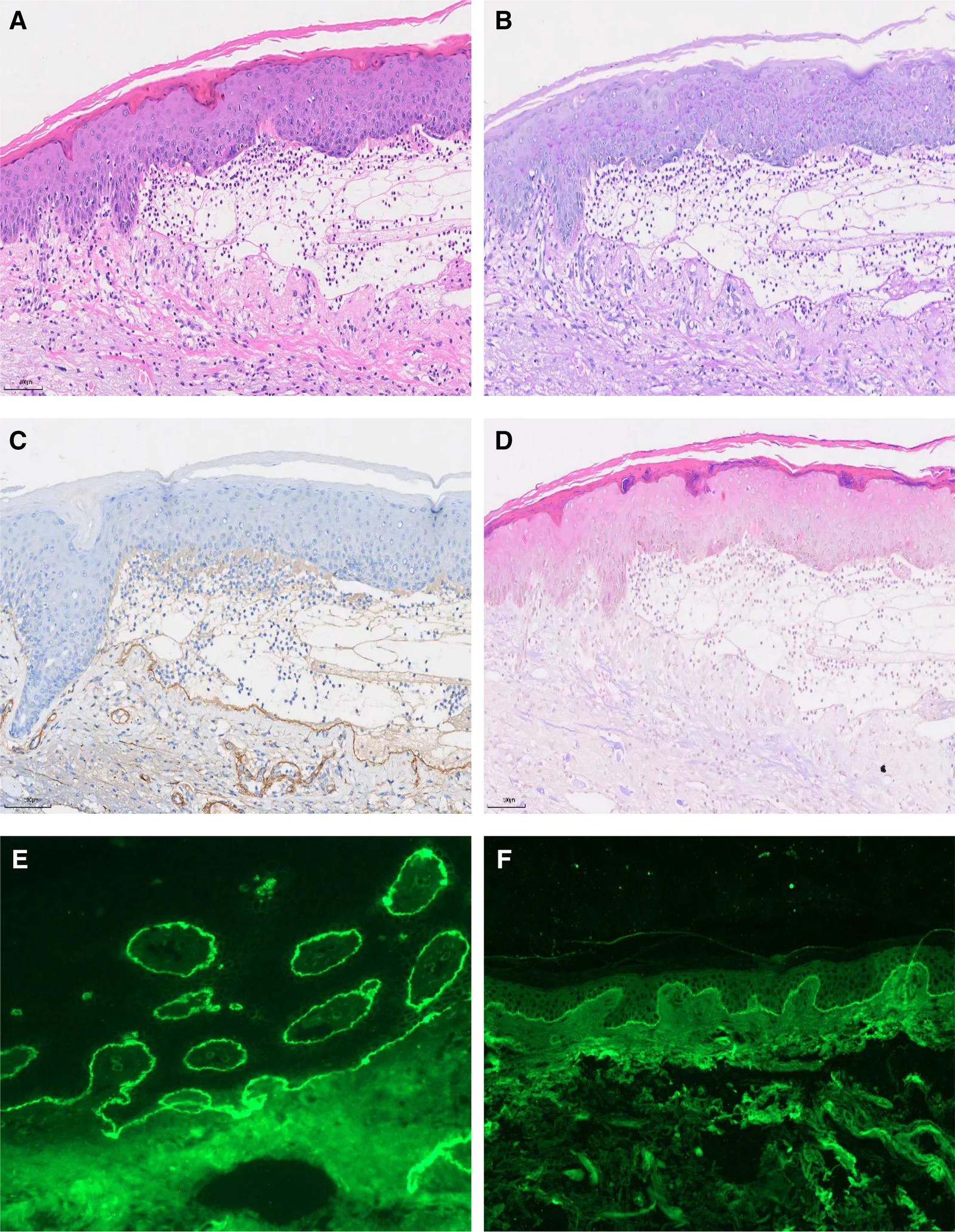
Histopathological findings at 200× magnification. (**A**) Hematoxylin and eosin staining revealed a cavity between the epidermis and dermis containing neutrophils. The superficial dermis exhibited edema, mucoid degeneration, and infiltration by lymphocytes and neutrophils. (**B**) Periodic acid-Schiff staining revealed fragmentation of the basement membrane zone and faint staining at the blister base. (**C**) Immunohistochemical staining revealed type IV collagen in the subepidermal blister floor. (**D**) Gram staining revealed no evidence of Gram-positive or Gram-negative organisms. (**E**) Direct immunofluorescence yielded positive results for complement C3. (**F**) Direct immunofluorescence revealed linear IgG deposition.

**Figure 3.**
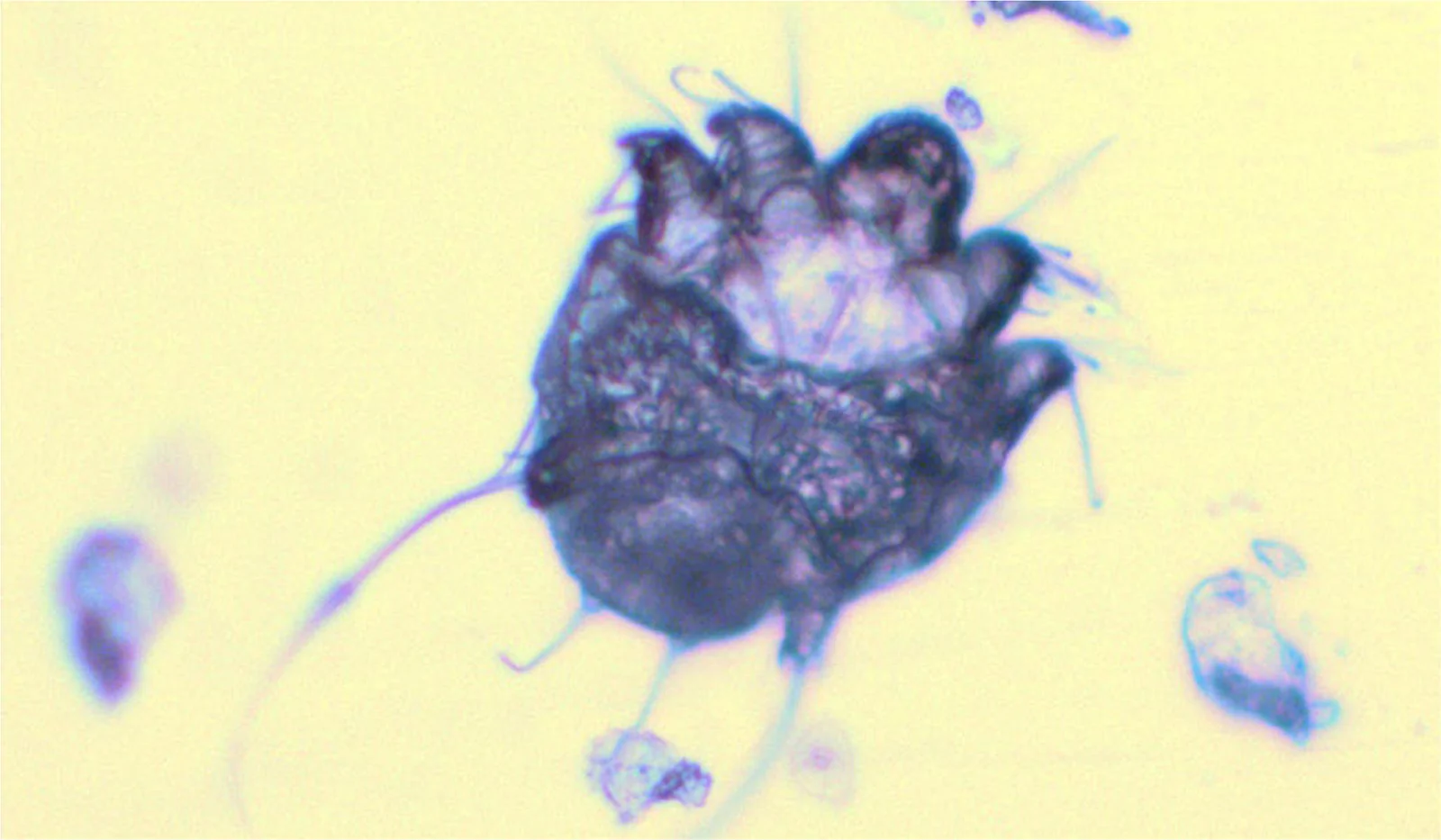
Microscopic findings. Scabies mites can be seen in microscopic images.

The patient received 40 mg/day of methylprednisolone initially. Upon BP diagnosis, dupilumab was added (600-mg loading dose, followed by 300 mg every 2 weeks). Rapid improvement occurred within 1 week, with blister resolution and fading erythema. Meanwhile, glucocorticoids were tapered and discontinued. For scabies, the patient received oral ivermectin (12.5 mg/week for 3 weeks) plus debridement and 10% sulfur ointment. After 30 days of combination therapy with dupilumab and ivermectin, he achieved significant resolution of truncal and hand lesions (Figure 1B and D). Given the high contagiousness of scabies, the patient was placed in isolation during hospitalization. His clothes underwent high-temperature sterilization, and his living environment was disinfected. Furthermore, family members with similar symptoms were also diagnosed with scabies at an external hospital and isolated for treatment. In October 2025, the patient was discharged after the second dose of dupilumab. Subsequently, he received the last dose of dupilumab. Follow-up continued until late December 2025, when the patient died due to pulmonary infection. This infection may have originated during his previous admission to an external hospital, but the detailed microbiological records were unavailable. Notably, no recurrence of skin lesions was observed before his death.

## DISCUSSION

Although cases of BP coexisting with NS have been sporadically documented, they still pose considerable diagnostic and therapeutic challenges due to clinical mimicry and a therapeutic dilemma.^8^^–^^10^ That is, BP often requires immunosuppression, whereas NS demands aggressive acaricidal therapy and infection control.^9^^,^^19^ For instance, Ye et al. reported a 70-year-old male patient with BP in whom NS and *Candida albicans* infection developed after glucocorticoid therapy.^10^ Additionally, Ran et al. reported a 73-year-old BP patient who developed scabies hyperkeratotic lesions on the glans penis during immunosuppressive therapy.^8^ Other similar cases that have been reported are summarized in Table 1.^9^^,^^20^^–^^22^ These cases suggest that glucocorticoid therapy can increase the susceptibility to scabies. Notably, authors of previous reports did not attempt to address glucocorticoid-related risks among the elderly,^8^^–^^10^^,^^20^^–^^22^ whereas the present case highlights dupilumab as a viable alternative. The authors of the present report have analyzed the patient’s clinical course and reviewed relevant literature to explore pathogenic mechanisms, identify diagnostic pitfalls, and discuss individualized management.

**Table 1:** Summary of cases of bullous dermatoses associated with scabies

Author, Year, and Reference	Case Characteristics
Gambichler et al. 2024^20^	A 4-month-old infant suffered from refractory scabies, which subsequently triggered BP.
Wang et al. 2023^9^​	An 89-year-old female patient suspected of having BP developed NS after receiving dexamethasone treatment.
Li et al.​ 2021^21^	Ten cases of BS were reported. It is suggested that for patients with blisters and poor response to steroids, the possibility of scabies should be considered for investigation.
Ashrafzadeh et al.​ 2000^22^	A 57-year-old woman with PV developed NS after receiving immunosuppressive treatment.

BP = bullous pemphigoid; NS = Norwegian scabies; PV = pemphigus vulgaris.

A mutually aggravating cycle can develop between BP and NS. First, intense pruritus and extensive skin damage in BP compromise the epidermal barrier, facilitating mite invasion and proliferation.^23^ Second, conventional BP treatments, such as systemic corticosteroids and immunosuppressants, further impair cellular immunity (the primary defense against scabies mites), enabling limited infestations to progress into NS.^24^ Furthermore, the dominant Th2 immune response in BP may relatively weaken the Th1 response, which is crucial for combating parasitic infections.^24^ Conversely, mite burrowing and resultant scratching can induce new blisters via the Koebner phenomenon in active BP patients.^6^ Additionally, scabies could further aggravate BP by increasing anti-BMZ antibodies.^10^^,^^20^ In the present case, NS emerged after corticosteroid therapy at an outside hospital, suggesting that BP-associated immunosuppression was the primary precipitating factor.

Diagnostically, hyperkeratotic scales and crusts of NS can mimic eczematized or secondary lesions of BP, and pruritus may be misattributed solely to BP activity.^23^ Additionally, conditions such as bullous scabies can clinically and histologically resemble pemphigoid.^6^^,^^23^ In such scenarios, serological testing for anti-BP180 and anti-BP230 antibodies is critical for confirming BP.^9^^,^^20^ This was exemplified in the study patient, whose crusts were initially misdiagnosed as BP-related eczema until microscopic examination confirmed scabies, underscoring the need to include NS in the differential diagnosis of immunosuppressed BP patients with atypical presentations or poor therapeutic response. Some key diagnostic points are summarized in Table 2.

**Table 2:** Differential diagnosis of bullous diseases

Disease	Cause	Lesion	Key Evidence
BP with NS	BP: autoimmunity; NS: scabies	BP: tense bullae, Nikolsky’s sign (–); NS: hyperkeratotic scales and crusts	BP: anti-BP180/230 antibodies (+); NS: find scabies
BS	Scabies	Tense bullae, Nikolsky’s sign (–)	Find scabies/anti-BP180/230 antibodies (–)
PV	Autoimmunity	Flaccid bullae, Nikolsky’s sign (+)	Anti-Dsg1/3 antibodies (+)

Anti-Dsg1/3 = anti-desmoglein 1/3; BP = bullous pemphigoid; BS = bullous scabies; NS = Norwegian scabies; PV = pemphigus vulgaris.

Long-term cumulative exposure to systemic glucocorticoids is significantly associated with severe infections, adverse prognosis, and increased mortality.^13^^,^^14^^,^^25^ This risk is particularly prominent in patients with BP who are elderly, have extensive skin barrier damage, and have multiple underlying diseases.^13^ Additionally, systemic glucocorticoids also cause other adverse reactions such as osteoporosis, myopathy, thromboembolism, and metabolic disorders.^11^^,^^15^ Glucocorticoid-induced sepsis and respiratory infections are the main causes of death.^25^ Therefore, the clinical guidelines recommend minimizing glucocorticoid exposure by optimizing local therapy and introducing glucocorticoid-sparing agents such as doxycycline, biologics (e.g., dupilumab), immunomodulators, and immunosuppressants in early treatment.^13^^,^^14^ Given that the patient developed a pulmonary infection during previous glucocorticoid therapy at another hospital, a “scabies-first” concomitant treatment strategy was implemented. In the early stage, dupilumab was introduced to reduce glucocorticoid dosage and avoid extensive immunosuppression.^12^^–^^15^ At the same time, ivermectin was combined for antiparasitic treatment, and local care and supportive treatment were provided to prevent secondary infections. The results reveal that dupilumab was effective within 1 week when combined with glucocorticoids, much faster than the median time of ∼2 months for its monotherapy.^26^ This integrated regimen controlled the skin lesions without rebound, revealing the feasibility of synchronized management under close monitoring. Ultimately, heightened recognition of this clinically complex and public health-relevant comorbidity is essential for early intervention and transmission control.

## References

[1] Nieto-Benito LM Suárez-Fernández R, 2026. The morbidity of systemic steroids: Analyzing its use in bullous pemphigoid. An Bras Dermatol 101(1): 501266. 10.1016/j.abd.2025.501266 PMC12809063 41506184

[2] Kim BR Lee KH Paik K Kim M Bae JM Choi CW Youn SW, 2025. Comorbid diseases in bullous pemphigoid: A population‐based case–control study. J Dermatol 52: 460–471. 10.1111/1346-8138.17577 39670559

[3] Akbarialiabad H, , 2025. Bullous pemphigoid. Nat Rev Dis Primers 11(1): 12. 10.1038/s41572-025-00595-5 39979318

[4] Huttelmaier J Benoit S Goebeler M, 2023. Comorbidity in bullous pemphigoid: Up-date and clinical implications. Front Immunol 14: 1196999. 10.3389/fimmu.2023.1196999 PMC10346857 37457698

[5] Asnawi V Maulida M Hidayati A Gunawan H Dwiyana RF Hindritiani R, 2023. Combination of crusted scabies with bullous scabies: A rare case. Int Med Case Rep J 16: 153–158. 10.2147/IMCRJ.S396234 PMC10018284 36936186

[6] Mauro A Colonna C Taranto S Garella V Castelletti F Giordano L Monzani NA Bernardo L, 2024. The hidden scabies: A rare case of atypical Norwegian scabies, case report and literature review. Ital J Pediatr 50(1): 7. 10.1186/s13052-023-01547-z PMC10795281 38233867

[7] Yang Y Shen H Liu ZH, 2023. Crusted scabies in an elderly patient. Am J Trop Med Hyg 109(6): 1209–1210. 10.4269/ajtmh.23-0202 PMC10793039 37748763

[8] Ran D Bao F Du D, 2024. Unique scabies presentation in a patient with bullous pemphigoid. Am J Trop Med Hyg 111(3): 455–456. 10.4269/ajtmh.24-0113 PMC11376171 39013382

[9] Wang X Liu Y Li J Bao F Chen M, 2023. Case of concurrence of bullous pemphigoid and Norwegian scabies. Parasite Immunol 45(3): e12969. 10.1111/pim.12969 36645298

[10] Ye Y Lv W Chai L Li Q Liu Z Xia X, 2026. Case report: Simultaneous presence of *Sarcoptes scabiei* and fungal elements in scrapings from a patient with bullous pemphigoid. Front Med (Lausanne) 13: 1773134. 10.3389/fmed.2026.1773134 PMC13006239 41877790

[11] Oren-Shabtai M Kremer N Lapidoth M Sharon E Atzmony L Nosrati A Hodak E Mimouni D Levi A, 2021. Treatment of bullous pemphigoid in people aged 80 years and older: A systematic review of the literature. Drugs Aging 38(2): 125–136. 10.1007/s40266-020-00823-5 33230804

[12] Silva JONd Silva RRe Ribeiro PVZ Farah PS Steglich RB, 2025. Efficacy and safety of dupilumab in patients with moderate-to-severe bullous pemphigoid: A systematic review and meta-analysis. An Bras Dermatol 100: 429–438. 10.1016/j.abd.2024.08.008 PMC12234199 40158906

[13] Powers CM Thakker S Gulati N Talia J Dubin D Zone J Culton DA Hopkins Z Adalsteinsson JA, 2025. Bullous pemphigoid: A practical approach to diagnosis and management in the modern era. J Am Acad Dermatol 92(6): 1337–1350. 10.1016/j.jaad.2025.01.086 39914667

[14] Borradori L, , 2022. Updated S2 K guidelines for the management of bullous pemphigoid initiated by the European Academy of Dermatology and Venereology (EADV). J Eur Acad Dermatol Venereol 36(10): 1689–1704. 10.1111/jdv.18220 35766904

[15] Liang G, , 2025. Dupilumab, corticosteroids and their combination for the treatment of bullous pemphigoid. An Bras Dermatol 100(2): 243–252. 10.1016/j.abd.2024.04.012 PMC11962819 39694724

[16] Kasperkiewicz M van Beek N Schmidt E, 2026. Autoimmune bullous diseases: Therapeutic update. Drugs 86(4): 485–506. 10.1007/s40265-026-02292-3 PMC13005878 41729378

[17] Harrell J Rubio XB Nielson C Hsu S Motaparthi K, 2019. Advances in the diagnosis of autoimmune bullous dermatoses. Clin Dermatol 37(6): 692–712. 10.1016/j.clindermatol.2019.09.004 31864451

[18] Lehman JS, 2026. Diagnosis of autoimmune blistering diseases: Dermatopathology and other laboratory updates. Dermatol Clin 44(2): 321–340. 10.1016/j.det.2026.01.011 41951330

[19] Angelo C Pedicelli C Provini A Annessi G Zambruno G Paradisi M, 2004. Successful treatment of Norwegian scabies with ivermectin in a patient with recessive dystrophic epidermolysis bullosa. Minerva Pediatr 56: 353–357. 15252385

[20] Gambichler T Mansour R Rothoeft T Schmidt E Doerler M Susok L, 2024. Association of recalcitrant scabies infestation and bullous pemphigoid in an infant. Dermatol Rep 16(2): 9751. 10.4081/dr.2024.9751 PMC11216140 38957636

[21] Li F-Z Jia M Chen K-J Ye Q Fang S, 2021. Bullous scabies: Clinical, dermoscopic, and pathologic characteristics of ten patients. Am J Trop Med Hyg 105(6): 1798–1802. 10.4269/ajtmh.21-0516 PMC8641337 34491217

[22] Ashrafzadeh S Layegh P, 2020. Crusted scabies in a patient with pemphigus vulgaris after treatment with rituximab and corticosteroids. JAAD Case Rep 6(8): 722–724. 10.1016/j.jdcr.2020.06.001 PMC7369456 32715061

[23] Cohen PR, 2017. Scabies masquerading as bullous pemphigoid: Scabies surrepticius. Clin Cosmet Investig Dermatol 10: 317–324. 10.2147/CCID.S145494 PMC5574693 28883737

[24] Bernard P Antonicelli F, 2017. Bullous pemphigoid: A review of its diagnosis, associations and treatment. Am J Clin Dermatol 18(4): 513–528. 10.1007/s40257-017-0264-2 28247089

[25] Chang TH Wu CY Chang YT Lyu YS Wu CY, 2023. Risk of serious infections in patients with bullous pemphigoid: A population-based cohort study. Acta Derm Venereol 103: adv5329. 10.2340/actadv.v103.5329 PMC10259460 37272362

[26] Abdat R, , 2020. Dupilumab as a novel therapy for bullous pemphigoid: A multicenter case series. J Am Acad Dermatol 83(1): 46–52. 10.1016/j.jaad.2020.01.089 32179082

